# The evaluation of pupil diameter by using Sirius before and after phacoemulsification in healthy, diabetic and hypertension patients

**DOI:** 10.1097/MD.0000000000033223

**Published:** 2023-04-21

**Authors:** Ali Simsek, Müslüm Toptan

**Affiliations:** a Department of Ophthalmology, Harran University, School of Medicine, Sanliurfa, Turkey.

**Keywords:** autonomic neuropathy, diabetes mellitus, phacoemulsification, pupil diameter, Sirius, systemic hypertension

## Abstract

Pupil size can affect the selection of the ablation region in refractive surgery and intraocular lens design in cataract surgery. Therefore, the evaluation of pupil diameter (PD), one of the anterior segment parameters, is an important component of ophthalmological examination.

**Material and methods:**

Seventy-one healthy patients, 64 patients with Systemic Hypertension (HT), and 65 patients with Diabetes Mellitus (DM) scheduled for phacoemulsification were included in the study. PD was measured before and one month after surgery using combined Scheimpflug-Placido disk topography (Sirius, CSO Inc.). Preoperative PD values of the groups were compared. The PD of the groups was compared in the 1st month after surgery. Then, preoperative and postoperative pupil diameter values of the groups were compared. The effect of the surgery on the change in pupil diameter (effect value) in the groups was also examined.

**Results:**

Pre- and postoperative PD only differed significantly between the healthy and DM groups (*P* = .039 and *P* = .045, respectively). PD decreased in all three groups after phacoemulsification. Pre-and postoperative PD differed significantly in the healthy group (4.78 ± 0.94 and 3.01 ± 0.48 mm, respectively, *P* < .05). Pre- and postoperative PD values also differed significantly (4.69 ± 0.84 and 2.95 ± 0.42 mm, respectively, *P* < .05). In the DM group, Pre- and postoperative PD also differed significantly in the DM group (4.38 ± 1.08 and 2.82 ± 0.43 mm, respectively, *P* < .05). The effect values of PD changes differed in the healthy, DM, and HT groups (1.95, 1.41, and 2.28, respectively). Phacoemulsification was observed to have a greater effect on PD change in HT patients.

**Conclusions:**

PD was smaller in DM patients than in the other groups. PD decreased in all three groups after phacoemulsification. This change should be remembered when planning cataract surgery for chronic metabolic patients.

## 1. Introduction

The evaluation of pupil diameter (PD), one of the anterior segment parameters, is an important component of ophthalmological examination. Pupil size can affect the selection of the ablation region in refractive surgery and intraocular lens (IOL) design in cataract surgery. At the same time, accurate measurement is essential in order to avoid adverse postoperative outcomes such as halos, glare, or poor night vision.^[1]^

In clinical practice, anterior segment imaging is traditionally performed using slit lamp biomicroscopy, although objective parameter evaluation is limited. Various devices including optical coherence tomography, ultrasonic biomicroscopy, Scheimpflug imaging, scanning slit tomography, and interferometry have been widely used for anterior segment evaluation in recent years.^[2]^ The Scheimpflug (Sirius) camera system is particularly valuable in terms of evaluating the anterior and posterior surface of the cornea, anterior chamber depth, PD, anterior chamber angle, and the iris and lens.^[3]^

PD evaluation in modern life is important in predicting patients’ visual outcomes.^[4]^ It is important for a better understanding of vision quality in pseudophakic patients to know that phacoemulsification surgery produces a change in PD. The diameter of the pupil is known to be associated with various visual functions in pseudophakic patients, such as glare,^[5]^ image alignment distance,^[6]^ binocular visual acuity,^[7]^ and near and distant visual acuity with multifocal IOL.^[8]^

Modern studies have reported that pupil shape and response frequently change following phacoemulsification surgery.^[9]^ A fixed dilated pupil emerges particularly after intracapsular and extracapsular cataract extraction.^[10]^ One study reported no correlation between pre- and postoperative PD.^[11]^

The surgical technique employed, the presence of a systemic disease affecting the eye, and the drugs used by the patient are all important in changes in PD and pupil shape occurring after phacoemulsification surgery.^[12]^ Diabetes mellitus (DM) is a chronic systemic disease characterized by impairment in carbohydrate, fat, and protein metabolisms. Diabetic retinopathy and diabetic autonomic neuropathy are recognized complications of diabetes. The emergence of these complications is associated with the duration of DM, and also with changes in the pupil.^[13]^ Hypertension (HT) causes microangiopathy through different mechanisms to those of diabetes. It leads to hypertensive retinopathy, retinal vascular occlusion, retinal microaneurysm, and ischemic optic neuropathy.^[14]^

The present study investigated the effects on PD of DM and HT, as causes of retinopathy, and examined PD values before and after phacoemulsification surgery in these patient groups. The presence of correlation between pre- and postoperative PD was also investigated.

## 2. Materials and Methods

### 2.1. Ethical considerations

The study was approved by Adiyaman University Medical Faculty Clinical Ethical Committee Date: 12.14.2014 and Number: 2014-10-02. All procedures were performed in compliance with the tenets of the Declaration of Helsinki.

### 2.2. Study design and data collection

This prospective study was performed at the Adiyaman University Medical Faculty Eye Diseases Clinic, Turkey, between 01.01.2015 and 01.12.2018.

Seventy-one healthy patients, 64 patients with HT, and 65 DM patients undergoing phacoemulsification and uncomplicated phacoemulsification surgery were included in the study. The calculated power (1-beta) was nearly 1, considering type I error (alfa) of 0.05, sample size of 62 to 65 in each group, effect size of 6.82 for the study and two-sided alternative hypothesis (H1).

Patients with ischemic optic neuropathy, other optic nerve diseases, or retinal artery occlusion, or with histories of glaucoma, cerebrovascular stroke or transient ischemic attack, iris neovascularization, iris atrophy, or pupil asymmetry were excluded from the study. Individuals with cataracts capable of preventing fundus examination and retinal pathology classification were also excluded. In addition, patients with myopic, hyperopic, and astigmatic refractive errors greater than ±1.0 D were also excluded. Patients with cataract cortical lens opacities greater than 2+, and with HT and DM were included in the study. Hypertensive retinopathy patients with mild thinning in the artery (Grade 1) were included in the study. Diabetic individuals were graded in terms of stage of non-proliferative diabetic retinopathy (NPDR) and were classified as diabetic with no NPDR, mild NPDR, moderate NPDR, or severe NPDR, based on the International Clinical Disease Severity Scale for diabetic retinopathy (DR).^[15]^ Only DR patients with no NPDR were included.

PD measurements were obtained before phacoemulsification and one month after surgery from the healthy individuals and HT and DM patients using the Combined Scheimpflug-Placido Disk System (Sirius, CSO Inc.). All measurements were performed by the same technician under darkened conditions. All measurements were taken between 14:00 and 17:00 hours in order to reduce diurnal corneal hydration variations to a minimum. Sirius is a novel topographic device that combines a monochromatic Scheimpflug camera capable of 360-degree rotation and a 22-ring Placido disk, yielding 25 radial sections from the cornea and anterior chamber. It provides information concerning the tangential and axial curvature of anterior and posterior surfaces of the cornea in a single section, and gives the global reactive power of the cornea, with biometric measurements of most intraocular structures. Once the screening is completed, the device yields PD values together with several anterior chamber parameters.

All operations were performed by the same experienced surgeon (AŞ), with topical anesthesia provided by proparacaine drops (Alcaine; Alcon, Istanbul, Turkey). A 2.4-mm clear corneal incision was made in the upper quadrant. Following viscoelastic injection (Viscoat; Alcon Surgical, Fort Worth, TX) an approximately 5.5-mm diameter continuous curvilinear capsulorhexis was performed. Cortical cleaving hydrodissection was carried out using balanced salt solution (Alcon Laboratories, Hemel Hempstead, UK). The nucleus was emulsified using torsional phaco technology (Centurion, Phacoemulsification System, Alcon, MI) with the freeze and cut method. Once the remaining cortex was cleansed with irrigation and aspiration was folded into the capsular bag, an acrylic, foldable, posterior chamber monofocal IOL (AcrySof, SA60AT; Alcon) was implanted in the capsular bag. Stromal hydration was applied to ensure wound integrity. Topical antibiotics and steroid drops were used postoperatively in all eyes. The topical antibiotic drops were applied four times daily for one week. The topical steroid drops were applied four times daily for one week and were subsequently gradually tapered over three weeks.

### 2.3. Statistical analysis

Statistical analyses were performed on SPSS 15.0 for Windows software (SPPS Inc., Chicago, IL). All data were expressed as mean ± standard deviation (SD). Normality for continuous variables in one group was determined using the Shapiro–Wilk test. Variables were normally distributed (*P* > .05), and the matched pairs *t* test was therefore applied to compare variables before and after surgery. Pearson’s correlation test was applied to determine correlations. The *P* values < .05 between variables were regarded as statistically significant. The effect sizes of surgery on the groups were calculated (effect size value *d* = t/√n or *d* = difference between means/standard deviation of the difference between means). The greater the effect size, the greater the change in PD after surgery.

## 3. Results

The healthy group consisted of 35 women (49.3%) and 36 men (50.7%) with a mean age of 65.27 ± 9.05. The HT group of 31 women (48.5%) and 33 men (51.6%) with a mean age of 63.03 ± 5.73, and the diabetic group consisted of 35 women (53.8%) and 30 men (46.2%) with a mean age of 64.05 ± 6.53. No statistical significance was observed between the group in terms of age or sex (*P* > .05) (Table 1).

**Table 1:** Demographic and PD of the groups.

Groups	N	Age (yr)	Preop PD (mm)	Postop PD 1st month (mm)	*P* *	*r (P*†/sign.)
Healthy	71	65.27 ± 9.05	4.78 ± 0.94	3.01 ± 0.48	<.001	0.32 (.007)
Diabetic	65	64.05 ± 6.53	4.38 ± 1.08	2.82 ± 0.43	<.001	0.15 (.223)
Hypertension	64	63.03 ± 5.73	4.69 ± 0.84	2.95 ± 0.42	<.001	0.42 (.001)

*P* **<** .05 (Significance of difference).

The difference between pre- and postoperative PD values in the hypertension group and diabetic group were statistically significant (*P* < .001).

PD = pupil diameter, *r* = Pearson Correlation Coefficient.

* Comparison of the group’s preop PD and postop PD values Paired Sample *t* test.

† Pearson correlation test.

**Figure 1.**
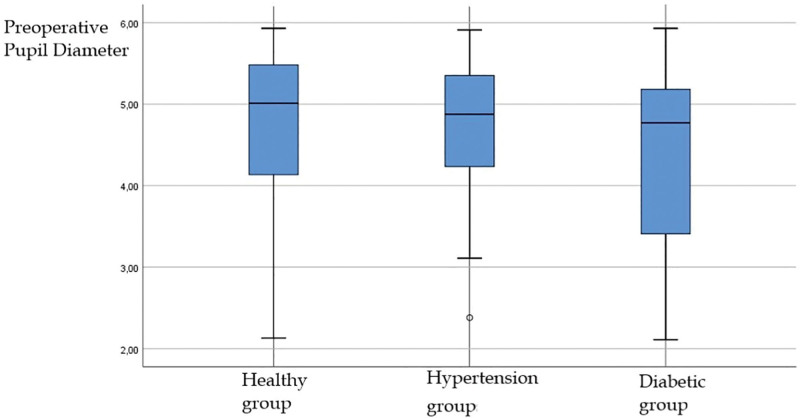
Preoperative pupil diameters in the Healthy, Systemic Hypertension, and Diabetic patient groups. The mean preoperative PD in the healthy group was 4.78 ± 0.94 mm. The difference between preoperative pupil diameter values in the hypertension group and diabetic group according to the healthy group was not statistically significant (*P* < .007). PD = pupil diameter.

**Figure 2.**
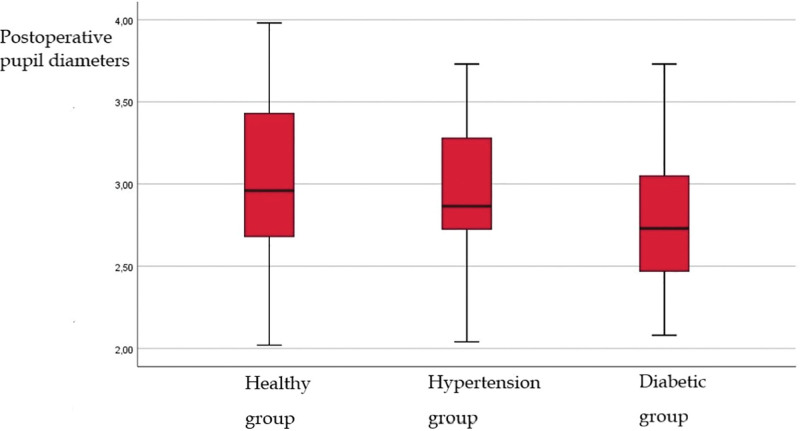
Postoperative pupil diameters in the Healthy Systemic Hypertension, and Diabetic patient groups. The mean preoperative PD in the HT group was 4.69 ± 0.84 mm. Postoperative PD was 2.95 ± 0.42 mm and decreased (*P* < .05). The difference between preoperative pupil diameter values in the hypertension group and diabetic group according to the healthy group was statistically significant (*P* < .05). HT = hypertension, PD = pupil diameter.

The mean preoperative PD in the healthy group was 4.78 ± 0.94 mm. Postoperative PD was 33.01 ± 0.48 mm and decreased (*P* < .05). Change in PD in the healthy group showed a positive correlation (*P* < .05). The mean preoperative PD in the HT group was 4.69 ± 0.84 mm. Postoperative PD was 2.95 ± 0.42 mm and decreased (*P* < .05). Change in PD in the HT group showed a positive correlation (*P* < .05). The mean preoperative PD in the DM group was 4.38 ± 1.08 mm. Postoperative PD was 2.82 ± 0.43 mm and decreased (*P* < .05). There was no correlation in the change in PD in DM group (*P* > .05). Demographic and PD of the groups are shown in Table 1. Preoperative PD of the groups is shown in Figure 1. Postoperative PD of the groups is shown in Figure 2.

Preoperative PD values were found to be different between groups with the One Way Anova test (*P* = .039). Postoperative PD values were found to be different between groups with the One Way Anova test (*P* = .045). Effect values were analyzed to determine the group in which the greatest change in PD occurred. The highest change within the healthy, DM, and HT groups was observed in the HT group (1.95, 1.41, and 2.28, respectively).

## 4. Discussion

PD decreased in all the groups in the present study compared to pre-phacoemulsification values. PD was smaller both before and after surgery in the diabetic patients compared to the other groups, but the effect of surgery on PD was greater in the HT group.

PD varies depending on brightness and various other factors. PD has an important effect on visual functions.^[16]^ PD and pupil shape have a direct effect on field depth and also on retinal illuminance. This then directly affects visual performance. Numerous studies involving PD have been published in the literature.^[17]^

The majority of systems used to measure PD in clinical practice employ infrared illumination to capture a pupillary image.^[18]^ The Scheimpflug camera is a contemporary system employed to visualize PD. The Scheimpflug camera system permits objective evaluation of the anterior and posterior corneal surfaces, PD, anterior chamber angle, the iris, and the lens.^[3]^ Pentacam (Oculus Optikgerate, Wetzlar, Germany) was the first device using a Scheimpflug camera in ophthalmological practice. After Pentacam, Galilei (Ziemer Group, Port, Switzerland) introduced a combined Scheimpflug camera and Placido disk topography system. New devices employing both a Scheimpflug camera and a Placido disk topography system include Sirius (Costruzione Strumenti Oftalmici, Tuscany, Italy) and TMS-5 (Tomey, Nagoya, Japan).^[19]^ A Sirius device was employed in the present study.

The present study investigated the effects of phacoemulsification surgery in patients with DM and HT, frequently encountered diseases that lead to retinopathy. Previous studies concerning changes in PD following phacoemulsification surgery can be found in the literature. These studies have investigated the effects on PD of multifocal IOLs,^[11]^ posterior chamber phacic IOLs,^[20]^ iris-fixated IOLs and DM.^[21]^ These studies have observed statistically significant changes in PD and pupil shape following phacoemulsification surgery, with mean decreases in postoperative PD between 11% and 13%. PD changes may be associated with increasing anterior chamber depth and volume following bulky crystalline lens removal. Another factor is the replacement of an average 4.4-mm thick lens with a thinner IOL.^[22]^ These varying parameters in the anterior segment can permit a decreased IOL volume and greater freedom of movement of the constrictor iris muscles, and this can reduce PD by producing full contraction of the iris muscles. A rapid reduction in (or even complete elimination of) such contractions following cataract may be a factor contributing to a rounder (less eccentric) pupil. In the present study, PD was significantly lower after surgery in the control, HT, and DM groups.

Previous studies have concentrated on the effect of systemic diseases on PD, particularly DM. We encountered no study investigating the effect of HT, which exerts systemic effects similar to DM. Diabetes is known to cause neuropathy as a result of the microangiopathy it produces. Diabetic neuropathy is one of the widespread and severe complications of DM.^[23]^ Diabetic autonomic neuropathy can manifest in all systems and tissues with involuntary-autonomic nervous control, and can affect the sympathetic and/or parasympathetic nerves.^[24]^ Autonomic neuropathies involving the eye include pupillary disorders, and decreased tear production and corneal sensitivity.^[25]^ Diabetes-related pupillary autonomic neuropathy almost always manifests itself as a pupil that is smaller than normal. Diabetic small pupils, with a damage to the sympathetic nerves, emerge as a result of disruption of the autonomic balance in favor of the parasympathetic nerves.^[26]^ This situation in diabetic patients may derive from more sensitive sympathetic iris innervation and a longer nerve pathway.^[27]^ The emergence of autonomic neuropathy in diabetic patients may be a lengthy process. However, subclinical autonomic neuropathy may be detected in 1 to 2 years following diagnosis of diabetes.^[28]^ The study group in the present study consisted of five-year non-proliferative DM patients. Previous studies have reported a smaller PD in DM patients compared to individuals without DM.^[29,30]^ Similarly in the present study, PD values were lower both pre- and postoperatively in the DM patients then in the control group. We attributed the low preoperative PD in the DM patients to diabetes-related pupillary autonomic neuropathy. Elliott and Carter^[31]^ reported a decrease in PD following cataract surgery in DM patients. Hayashi and Hayashi^[32]^ reported that PD decreased on the third day postoperatively, but returned to close to preoperative values in 1 month. In the present study, PD decreased significantly in the first month postoperatively compared to preoperative values. We attribute this change to increasing anterior chamber depth following removal of the bulky crystalline lens, and to freer contraction of the iris muscles as a result of greater freedom of movement.

In contrast to DM, HT results in a microangiopathy that causes physical changes in blood vessels, such as thickening of the elastic lamina in the vascular wall and hyaline changes. HT-related changes in ocular tissue that may be directly observed in blood vessels include hypertensive retinopathy, retinal vascular occlusion, retinal macroaneurysm, and ischemic optic neuropathy. HT can exacerbate the vision-threatening effects of DR and has been identified as a risk factor for the development of age-related macular degeneration.^[14]^ PD decreased significantly following phacoemulsification in the HT group in this study. Both pre- and postoperative PD values were significantly lower in the HT group compared to the control group, but there was no statistically significant difference from the control group. The effect of phacoemulsification surgery on PD change was greater in the HT group than in the other two groups. We encountered no previous study of PD in HT patients. We think that these changes may be associated with impairment of autonomic balance in favor of sympathetic nerves in association with damage in the sympathetic nerves, similarly to diabetes, in the HT group.

The limitations observed during this study can be listed as follows. Patients with other higher stages of retinopathy related to DM and HT and treated for retinopathy were not included. DM and HT patients within the first 5 years of the disease were included in the groups. It was not possible to observe their effects on PD in the following years. Only patients without NPDR were included in the DM group, and only Grade 1 patients in the HT group. Therefore, we do not know the effects of other degrees of DM and HT-related retinopathy on PD. We believe that new studies are needed on the effects of phacoemulsification surgery on PD in patients with advanced HT and DM.

## 5. Conclusion

We provide evidence that the short-term clinically feasible Scheimpflug-Placido Disc System can be used to evaluate neural dysfunction associated with diabetes and hypertension. More importantly, the results show that eye changes begin in early diabetic and hypertensive patients, which indicates that Pupillometry can measure nerve abnormalities in these individuals.

It is very important to understand PD and surgery-related changes in HT and DM patients this time when intensive efforts are being made to achieve better visual outcomes following cataract surgery. The ability to predict changes following surgery will be of great assistance in terms of patients’ visual expectations and accurate lens selection.

Since there is a risk of developing vision loss due to serious vascular disorders in individuals with DM and HT, it is important to monitor the pupil diameter. PD may be a parameter in the treatment and follow-up of these patients. In addition, depending on the stage of the disease, it can be a parameter to evaluate the extent to which cataract surgery is beneficial on visual quality. But future studies are needed.

This study is an authentic one, and further studies should be conducted by increasing the number of samplings, including groups having other stages of DM and HT, and increasing the number of the applied parameters. And the information to be obtained in these studies will guide our diagnosis and treatment in the future.

## Author contributions

**Data curation:** Ali Simsek, Müslüm Toptan.

**Formal analysis:** Ali Simsek, Müslüm Toptan.

**Investigation:** Ali Simsek.

**Methodology:** Ali Simsek, Müslüm Toptan.

**Project administration:** Ali Simsek.

**Visualization:** Ali Simsek.

**Writing –original draft:** Ali Simsek, Müslüm Toptan.

**Writing –review & editing:** Ali Simsek.
